# Distribution of perceived weight stigma and its psychological impact on obese people in Saudi Arabia

**DOI:** 10.1016/j.jsps.2023.101763

**Published:** 2023-09-01

**Authors:** Wafi F Albalawi, Joud Albaraki, Sereen Alharbi, Nouf Ababtain, Reema Enad Aloteibi, Ali Saleh Alsudais, Jafar Jamjoom, Meshal Alaqeel

**Affiliations:** a Department of Community Health Sciences, College of Applied Medical Sciences - King Saud University, Saudi Arabia; b College of Medicine - King Saud bin Abdul-Aziz University for Health Sciences, Saudi Arabia; c Department of Mental Health, Ministry of The National Guard - Health Affairs, Riyadh, Saudi Arabia; d King Abdullah International Medical Research Center, Riyadh, Saudi Arabia

**Keywords:** Weight stigma, Stress, Obesity, Health, Discrimination

## Abstract

**Background:**

Worldwide, obesity prevalence has nearly tripled since 1975, with about 13% of adults being obese and about 39% overweight. Overweight and obese persons are vulnerable to frequent stigmatization and discrimination because of their weight, an issue that is barely discussed in the medical literature. In Saudi Arabia, the prevalence of obesity is 36%. However, there is no available data on the prevalence of perceived weight stigma among obese people. Therefore, this study aims to (a) determine the Distribution of perceived weight stigma among obese people, (b) identify the major sources of stigma, and (c) determine the psychological impact of perceived weight-based stigma on obese people in Saudi Arabia.

**Methods:**

This is a cross-sectional study conducted in Saudi Arabia using an online questionnaire that includes Stigmatizing Situations Inventory Scale (SSI) and Perceived Stress Scale (PSS).

**Results:**

1341 people participated in the study, of which 819 (61%) were females and 522 (39%) were male. Of all, 62 (5%) were underweight, 357 (27%) were normal weight, and 922 (69%) were overweight or obese. Participants in the overweight/obese category scored higher on average in every SSI item than did their counterparts in the underweight and normal weight categories, indicating higher levels of stigma among overweight and obese participants. The major sources of stigma for overweight and obese participants were identified based on the mean of participant responses to each item. These were: assumption about overeating or binge eating (mean response ± SD 2.80 ± 3.01), children's comments (2.22 ± 2.58), being stared at in public (2.18 ± 2.83) and being singled out as a child (2.05 ± 2.67).

**Conclusion:**

Parallel with the literature, our findings indicate a high prevalence of weight stigma in Saudi Arabia which can have negative psychological implications on obese people.

## 1 Introduction

Worldwide, obesity prevalence has nearly tripled since 1975, with about 13% of adults being obese and about 39% overweight (World Health Organization, 2021). Despite the adverse effects overweight and obesity can have on one's health, more people have these conditions today than ever before in history (World Health Organization, 2021). In Saudi Arabia, the prevalence of obesity is 36% (Althumiri et al., 2021). Importantly, obesity is a significant risk factor for illness and death; it is associated with diabetes, hypertension, hyperlipidemia, obstructive sleep apnea, and osteoarthritis. Moreover, with increases in life expectancy, obesity is causing more years of disability. Hence, the increasing cost of obesity and its sequelae will strain the resources of governments and individuals alike (Bray, 2003, Cawley and Meyerhoefer, 2012). Overweight and obese persons are vulnerable to frequent stigmatization and discrimination because of their weight, an issue that is barely discussed in public health interventions and the medical literature(DePierre and Puhl, 2012). Such negative attitude toward people who are perceived to have excess weight is referred to as weight stigma. Sources of weight stigmatization toward obese persons have been identified in the literature and include employers, healthcare providers, educators, peers, family members, and the media (Puhl and Brownell, 2001, Puhl and Heuer, 2009). A study conducted in the U.S found that 72% of participants rated family members as the most frequent source of weight stigma, often in weight-based teasing, name-calling, and inappropriate comments. The majority of participants also named friends (60%) and spouses (47%) as perpetrators of weight bias (Puhl and King, 2013).Table 1.

**Table 1:** Descriptive analysis (percentage and count) of demographic and socio-economic variables according to BMI.

**Variable**		**BMI Group**		
	Underweight	Normal weight	Overweight	Total
**Gender**				
Male	14.52% (9)	30.25% (1 0 8)	43.93% (4 0 5)	38.93% (5 2 2)
Female	85.48% (53)	69.75% (2 4 9)	56.07% (5 1 7)	61.07% (8 1 9)
**Age**				
18–24	87.1% (54)	63.31% (2 2 6)	40.78% (3 7 6)	48.92% (6 5 6)
25–29	3.23% (2)	15.13% (54)	16.59% (1 5 3)	15.59% (2 0 9)
30–34	4.84% (3)	9.8% (35)	12.15 % (1 1 2)	11.19% (1 5 0)
35–39	1.61% (1)	4.48% (16)	10.41% (96)	8.43% (1 1 3)
40–44	0% (0)	1.96% (7)	8.79% (81)	6.56% (88)
45–49	0% (0)	0.84% (3)	4.45% (41)	3.28% (44)
>50	3.23% (2)	4.48% (16)	6.83% (63)	6.04% (81)
**Region**				
Northern Region	14.52% (9)	13.73% (49)	9.33% (86)	10.74% (1 4 4)
Eastern Region	19.35% (12)	16.81% (60)	22.34% (2 0 6)	20.73% (2 7 8)
Central Region	24.19% (15)	31.09% (1 1 1)	25.92% (2 3 9)	27.22% (3 6 5)
Western Region	27.42% (17)	23.53% (84)	22.02% (2 0 3)	22.67% (3 0 4)
Southern Region	14.52% (9)	14.85% (53)	20.39% (1 8 8)	18.64% (2 5 0)
**Marital Status**				
Single	91.94% (57)	75.91% (2 7 1)	56.83% (5 2 4)	63.53% (8 5 2)
Married	6.45% (4)	22.97% (82)	39.48% (3 6 4)	33.56% (4 5 0)
Divorced	1.61% (1)	1.12% (4)	3.15% (29)	2.54% (34)
Widowed	0% (0)	0% (0)	0.54% (5)	0.37% (5)
**Highest Level of Education**				
Elementary	0% (0)	0% (0)	0.22% (2)	0.15% (2)
Secondary	0% (0)	0.285 (1)	0.98% (9)	0.75% (10)
High school diploma	38.71% (24)	31.09% (1 1 1)	24.84% (2 2 9)	27.14% (3 6 4)
Associate degree	3.23% (2)	7% (25)	8.89% (82)	8.13% (1 0 9)
Bachelor's degree	54.84% (34)	52.94% (1 8 9)	54.01% (4 9 8)	53.77% (7 2 1)
Graduate degree	3.23% (2)	8.68% (31)	11.06% (1 0 2)	10.07% (1 3 5)
**Employment Status**				
Employed	14.52% (9)	26.05% (93)	39.26% (3 6 2)	34.6% (4 6 4)
Unemployed	12.9% (8)	15.69% (56)	16.05% (1 4 8)	15.81% (2 1 2)
Student	70.97% (44)	52.94% (1 8 9)	36.66% (3 3 8)	42.58% (5 7 1)
Retired	0% (0)	3.08% (11)	4.34% (40)	3.8% (51)
Other	1.61% (1)	2.24% (8)	3.69% (34)	3.21% (43)
**Income**				
Less than 5000 SR	19.35% (12)	20.73% (74)	16.81% (1 5 5)	17.97% (2 4 1)
5000–––10,000 SR	16.13% (10)	20.17% (72)	20.61% (1 9 0)	20.28% (2 7 2)
10,000–––15,000 SR	16.13% (10)	16.25% (58)	18% (1 6 6)	17.45% (2 3 4)
More than 15,000 SR	29.03% (18)	29.41% (1 0 5)	31.45% (2 9 0)	30.8% (4 1 3)
Prefer not to say	19.35% (12)	13.45% (48)	13.12% (1 2 1)	13.5% (1 8 1)

Overweight and obese employees also face frequent discrimination in the workplace. Studies have demonstrated that employers often do not prefer to hire obese people as they are assumed to be less self-disciplined, thought more likely to have poor supervisory skills, and perceived to lack a professional appearance (Puhl and King, 2013). As a result, obese people are likely to earn less than their non-obese counterparts (Judge and Cable, 2011, Rothblum et al., 1988).

In healthcare settings, physicians' communication is affected by how they perceive their patients; for example, physicians are more likely to engage with patients whom they believe are adherent and satisfied (Rothblum et al., 1988). Many healthcare providers hold strong negative attitudes and stereotypes about people with obesity, and there is considerable evidence that such attitudes influence perceptions, judgment, interpersonal behavior, and decision-making. In particular, these attitudes and the common belief that overweight patients are lazy and less compliant may impact physicians' care, for example in spending less time with obese patients, recommending a diet even if the patient did not discuss weight loss, and blaming unrelated physical problems on weight (Rothblum et al., 1988).

The social acceptability of weight stigma is readily apparent through the media's negative depiction of overweight and obese persons. In both child and adult entertainment, overweight individuals are more likely to be cast as minor characters, targets of ridicule and humor, and depicted engaging in stereotypical behaviors (e.g., eating or bingeing on unhealthy foods); they are also less likely to be portrayed as having romantic partners or interactions with friends(Ashmore et al., 2008, Himes and Thompson, 2007).

Such fat shaming is an ongoing issue in modern society, as studies have shown an increase in the prevalence of weight stigma worldwide. A study comparing national differences in prejudice against obese people showed a 66% increase in prevalence in the United States over the last decade. The study attributed this stigmatization to the assumption that obese individuals are accountable for possessing said negative attribute or their weight gain (Faith et al., 2002).

Exposure to weight stigmatization contributes to a range of adverse psychological outcomes, including poor body image, low self-esteem, social isolation, and risk of depression and anxiety (Puhl and King, 2013). Weight bias may also reinforce unhealthy behaviors that contribute to obesity. Adults who report weight stigmatization engage in more frequent binge-eating behaviors, are more likely to be diagnosed with Binge Eating Disorder and are more likely to engage in maladaptive eating patterns and to exhibit eating disorder symptoms (Gray et al., 2008, Storch et al., 2006). Weight-based victimization has also been associated with reduced physical activity and increased cardiovascular risk (Alenazy R et al., 2021; Crandall et al., 2001); for example, overweight youth victimized by their peers are less likely to participate in physical activities and physical education classes (Storch et al., 2006).

In Saudi Arabia, the prevalence of obesity is 36%; however, the prevalence, causes, and consequences of weight-based stigma have received little or no attention from the scientific community. A recent 2020 study conducted in Saudi Arabian hospitals showcased that almost two-thirds of participants, half of whom were obese, have experienced some negative behavior aimed at them due to their weight gain (Alenazy R et al., 2021). There remains need for scientific research to identify the magnitude of the problem, and to further implement changes both at the level of healthcare providers and among the general public. Therefore, our study aimed to determine the distribution of perceived weight stigma among obese people, identify the major sources of stigma, and determine the psychological impact of perceived weight-based stigma on obese people in Saudi Arabia.

## 2 Methods

### 2.1 Study design

This cross-sectional study was conducted using an electronic-based questionnaire distributed online through social media platforms (e.g., Twitter, WhatsApp, Telegram). The questionnaire is composed of three parts: demographic data, the Stigmatizing Situations Inventory Scale-Brief (SSI-B)^18^, and the Arabic version of the Perceived Stress Scale (Vartanian, 2015). The collected demographic data included: gender, age, region, marital status, level of education, employment status, and income. The SSI-B is a 10-item scale used to measure perceived weight stigma; the items include situations a person may encounter due to their weight with a scoring that measures frequency from 0 to 9, with 0 as never and 9 as daily. The Perceived Stress Scale (Chaaya et al., 2010) is a stress assessment instrument composed of ten questions with answers on a scale from 0 to 4, with 0 as never and 4 as very often.

### 2.2 Scale translation and validation

A back-to-back translation of the Stigmatizing Situations Inventory Scale-Brief (SSI-B) from English to Arabic was conducted. We then performed a Cronbach's alpha test to measure the internal consistency of items and the reliability of the Arabic scale. The result showed high consistency and a reliable score of 0.9025, **(Appendices A).**

### 2.3 Data collection

Data was collected from January 2022 to March 2022. The inclusion criteria for the study were individuals ≥ 18 years old and Saudi. Although the study targeted obese people with BMI ≥ 30, other weight groups were also included and were used mainly for comparison purposes. Non-Saudi nationals were excluded from the study due to the possibility of occurring/existing discrimination and racism targeting non-Saudis, which may cause biased outcomes.

### 2.4 Statistical analysis

The statistical software packages Stata version 23 and IBM SPSS version 22 were used to obtain descriptive statistics, including frequency distribution and percentages, and to perform further analyses such as linear regression, principal component analysis, and multinomial logistic regression analysis. IRB approval was obtained and participant consent was taken prior to completing the questionnaire.

## 3 Results

A total of 1,341 individuals participated in the study, of which 61.07% (n = 819) were female and 38.99% (n = 522) were male. Participants were categorized by age into seven groups: 18–24 years (48.92%, n = 656), 25–29 years (15.59%, n = 15), 30–34 years (11.19%, n = 150), 35–39 years (8.43%, n = 13), 40–44 years (6.56%, n = 88), 45–49 years (3.28%, n = 44), and greater than 50 years (6.04%, n = 81). As for region, the largest fraction of participants (27.22%, n = 365) were from the central region, followed by 22.67% (n = 304) from the western region, 20.73% (n = 278) from the eastern region, 18.64% (n = 250) from the northern region, and 10.74% (n = 144) from the southern region.

For marital status, 63.53% (n = 852) were single, 33.56% (n = 450) married, 2.54% (n = 34) divorced, and 0.37% (n = 5) widowed. Concerning participants’ highest level of education, about half stopped at a bachelor’s degree (53.77%, n = 721), followed by high school with 27.14% (n = 364), graduate degree 10.07% (n = 135), associate degree 8.13% (n = 109), secondary school 0.75% (10), and finally elementary school 0.15% (n = 2). As regards employment status, the greatest proportion were students (42.58%, n = 571), followed by employed at 34.6% (n = 464), unemployed at 15.81% (n = 212), retired at 3.8% (n = 51), and other at 3.21% (n = 43). As for income status, the largest fraction reported their income as more than 15,000 SR (30.80%, n = 413), followed by an income of 5,000 to 10,000 SR at 20.28% (n = 272), income between 10,000 to 15,000 SR at 17.97% (n = 241), income less than 5,000 SR at 17.45% (n = 234), and finally 13.12% (n = 121) of participants preferred not to share their income status.

Participants were classified into three different weight groups based on their Body Mass Index (BMI) levels: those with BMI less than 18.5 were classified as underweight, those with a BMI in the range of 18.5–24.9 were classified as normal, and those with BMI over 25 were classified as overweight/obese. As shown in Table 2, out of 1341 participants, 68.75% (n = 922) were overweight or obese, 26.62% (n = -357) were of normal weight, and finally 4.62% (n = 62) were underweight.

**Table 2:** Distribution of stigma across BMI groups.

**BMI Group**	**Statistic**	**Stigmatizing Situations Inventory Scale-Brief (SSI-B)**									
		Being singled out as a child by a teacher, school nurse, etc., because of your weight	Being stared at in public	Children loudly making comments about your weight to others	Having a doctor recommend a diet, even if you did not come in to discuss weight loss	Having a romantic partner exploit you, because she or he assumed you were ‘desperate’ and would put up with it	Overhearing other people making rude remarks about you in public	Not being hired because of your weight, shape, or size	Having family members feel embarrassed by you or ashamed of you	Having people assume you overeat, or binge eat because you are overweight	Being glared at or harassed by bus passengers for taking up ‘too much’ room
**Underweight**	**N**	62	62	62	62	62	62	62	62	62	62
	**Mean**	0.90	1.00	0.94	0.39	0.65	0.66	0.40	0.60	0.44	0.55
	**SD**	2.07	2.18	2.22	1.19	1.73	1.65	1.64	1.57	1.33	1.74
**Normal weight**	**N**	357	357	357	357	357	357	357	357	357	357
	**Mean**	0.75	0.75	0.75	0.37	0.34	0.35	0.17	0.41	0.79	0.27
	**SD**	1.79	1.74	1.69	1.25	1.19	1.06	0.97	1.38	1.63	1.12
**Overweight/obese**	**N**	922	922	922	922	922	922	922	922	922	922
	**Mean**	2.05	2.18	2.22	1.87	0.99	1.19	0.70	1.28	2.80	1.16
	**SD**	2.67	2.83	2.58	2.22	2.08	2.01	1.70	2.32	3.01	2.09
**Total**	**N**	1341	1341	1341	1341	1341	1341	1341	1341	1341	1341
	**Mean**	1.65	1.74	1.77	1.40	0.80	0.94	0.54	1.01	2.16	0.89
	**SD**	2.51	2.63	2.45	2.08	1.89	1.83	1.56	2.12	2.81	1.91

Participants in the overweight/obese category scored higher on average in every SSI item than did their counterparts in the underweight and normal weight categories, indicating higher levels of stigma among overweight and obese participants. The major sources of stigma for overweight and obese participants were identified based on the mean of participant responses to each item. These were: assumption about overeating or binge eating (mean response ± SD 2.80 ± 3.01), children's comments (2.22 ± 2.58), being stared at in public (2.18 ± 2.83) and being singled out as a child (2.05 ± 2.67).

### 3.1 Principal component factor analysis

To increase the interpretability of the Stigmatizing Situations Inventory Scale, we conducted principal component factor analysis. Items were retained within a factor if they scored 0.30 or above (Kline, 1993), and the Kaiser-Guttman rule was applied for factor retention (Kaiser, 1974), which yielded just one dimension with an eigenvalue greater than 1 **(Appendices B).** This dimension was used in further analyses as the new variable stigma. We additionally combined all items of Perceived Stress Scale (PSS) together as a second new variable, stress.

After creating the two new variables stress and stigma, we applied regression analysis to better understand the relationship between them (Table 3). This revealed that stigma is a positive predictor of stress. As indicated by the coefficient, for every unit increase in the stigma measure, stress increases by 0.656; this implies that participants that are more stigmatized are also more stressed.

**Table 3:** Linear regression analysis of stigma in relation to stress.

Because our data is categorical, we further examined the association of stigma and stress in different BMI groups using multinominal logistic regression. The baseline or reference category was the underweight group. As shown in Table 4, compared to the underweight group, stigma is significantly associated with being overweight/obese (coef = 0.435, *p* = 0.000). However, stress is negatively associated with being overweight/obese (coef; −0.037, *p* = 0.024). In the normal-weight group, stress and stigma are negatively correlated but do not exhibit a significant effect on participants (*p* greater than 0.05). In other words, stress and stigma do not truly affect normal-weight participants. We can also conclude that overweight/obese participants experience less stress and more stigma compared to normal-weight people. Furthermore, the odds ratio of stress for overweight compared to underweight is exp(-0.037) = 0.96, which implies that those overweight are about 0.96 times less stressed compared to those underweight. However, the corresponding odds ratio of stigma is exp(0.435) = 1.54, indicating that those overweight are about 1.54 times more stigmatized compared to those underweight.

**Table 4:** Multinomial logistic regression of stigma and stress according to BMI group.

**BMI Group**	**Coef.**	**Std. Err.**	**Z**	**P > z**	**95% Conf.**	**Interval**
**Underweight**	(Base outcome)					
**Normal weight**						
Stress	−0.018	0.017	−1.060	0.290	−0.051	0.015
Stigma	−0.187	0.115	−1.620	0.105	−0.413	0.039
Constant	2.055	0.522	3.940	0.000	1.033	3.078
**Overweight & Obese**						
Stress	−0.037	0.016	−2.250	0.024	−0.068	−0.005
Stigma	0.435	0.104	4.200	0.000	0.232	0.638
Constant	3.862	0.500	7.730	0.000	2.882	4.842

We additionally investigated whether there is a gender difference in stigma and stress. We found insignificant gender difference in stigma (*p* = 0.26), indicating that men and women experience the same level of stigma. However, a significant difference exists in stress, with males having lower mean stress than females (24.86 + 8.32 vs. 28.71 + 0.28, *p* = 0.000), indicating that women experience more stress compared to men.

We further examined differences in stress and stigma according to income group. A significant mean effect of income group was found for stigma (F 2.46, *p* less than 0.05), illustrating those participants earning less than 5000 SR (0.33 ± 2.49), more than 15,000 SR (-0.13 ± 2.21), or that prefer not to say (-0.26 ± 2.18) experience less stigma compared to the middle-income groups. Similarly, a statistically significant difference was observed in stress among participants with diverse income levels (F 6.33, *p* less than 0.05), with those having lower incomes scoring higher on stress. These findings indicate that stress is inversely associated with income.

A similar examination of differences according to education group revealed no significant mean effect of education group in stigma (F 0.77, *p* greater than 0.05), indicating that people perceive the same level of stigma regardless of education level. However, a significant mean effect was identified for stress (F 2.83, *p* less than 0.05), indicating that stress is affected by education level. In particular, participants with secondary education (M = 30.2 ± 3.61) experience the most stress compared to other educational groups, followed by high school (M = 28.04 ± 8.49), associate degree (M = 27.25 ± 7.56), bachelor’s degree (M = 27.18 ± 8.53), elementary school (M = 26.0 ± 5.65), and finally those with a graduate degree (M = 24.98 ± 8.90).

## 4 Discussion

The aim of this study was to determine the distribution of perceived weight stigma among obese people, identify the major sources of stigma, and determine the psychological impact of perceived weight-based stigma on obese people in Saudi Arabia. The findings suggest that first, the majority of overweight and obese people have experienced stigma at least once in their lifetime, and the major sources of stigma are assumption of overeating or binge eating, children’s comments, public staring, and being singled out as a child; these results parallel many studies from the literature(Friedman et al., 2008, Friedman et al., 2005, Major and O’Brien, 2005, Tomiyama, 2014). For example, multiple prior reports have highlighted sources such as small chairs, small medical equipment, and “inappropriate assumptions about the obese person,” which were experienced by as many as 97% of participants in the study of Friedman et al.(Friedman et al., 2005, Friedman et al., 2008). Moreover, in a study of gastric bypass and overweight patients, identified “comments from children about obese person’s weight” as the most common source of stigma, which is also the second most common source in this study (Friedman et al., 2008).

**Fig. 1:**
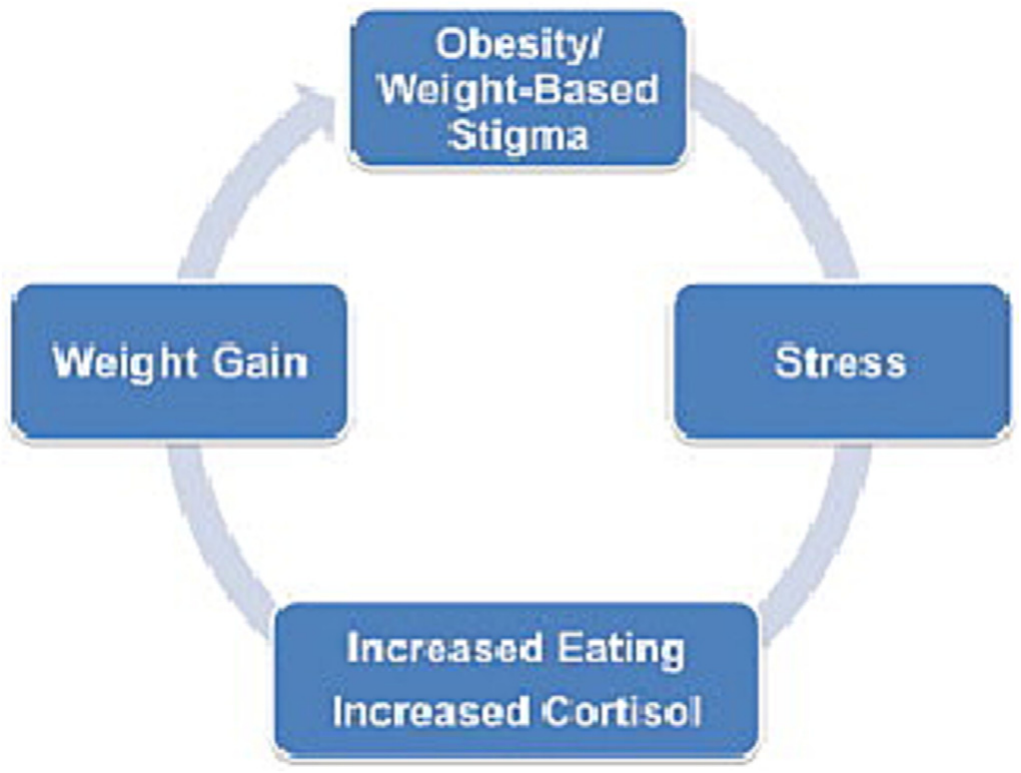
Cyclic Obesity/Weight-Based Stigma (COBWEBS) Model. A. Janet Tomiyama, Appetite, 2014.

Second, we identified a strong linear relationship between stigma and stress, indicating that people who experience weight-based stigma are most likely going to develop stress. The impact of weight-based stigma on stress level has been well established (DePierre and Puhl, 2012, Friedman et al., 2008, Gray et al., 2008, Himes and Thompson, 2007, Judge and Cable, 2011, Puhl and Brownell, 2001, Puhl and Heuer, 2009, Puhl and King, 2013, Rothblum et al., 1988, Tomiyama, 2014); in fact, many studies have suggested that stress resulting from weight-based stigma leads people to participate in unhealthy behaviors such as eating unhealthy food and avoiding exercise, which in turn makes them susceptible to further weight gain (Major and O’Brien, 2005, Tomiyama, 2014). The Cyclic Obesity/Weight-Based Stigma (COBWEBS) model (Fig. 1) illustrates this feedback effect; it suggests that when people experience weight-based stigma, they start developing stress, which in return impacts their eating behavior and plasma levels of cortisol—a stress hormone that promotes fat storage. This process has the net effect of promoting weight gain, which then exposes individuals to more experiences of weight stigma, perpetuating the cycle (Gaskin et al., 2013, Tomiyama, 2014).

Third, our multinomial logistic regression of stress according to weight group found that overweight people perceive more stigma but experience less stress when compared to other BMI groups (Table 3). This could be explained by the characteristics of the participants, as most were in the 18–24 age bracket. Individuals in this age group could possibly experience more stress regardless of BMI level due to factors such as school, financial difficulties, self-perception, and excessive mobile device use. For example, one study investigated the differences in self-rated health (SRH) among young adults in a nationwide study of over 13,500 participants; they found that irrespective of actual BMI, those who perceived themselves as underweight or overweight reported poorer health, in contrast to those that thought they were of normal weight (DePierre and Puhl, 2012, Idema et al., 2019). Therefore, young adults’ perceptions of themselves can influence how they rate their health, which in turn could influence the level of stress they experience.

There are mixed findings in the literature as to whether males and females experience different rates of weight-based stigma and related discrimination. Many studies have suggested that women experience greater risk of weight-based stigma compared to men (Gaskin et al., 2013, Idema et al., 2019), but some have suggested there to be no gender difference in weight-based stigma (Hebl and Turchin, 2005; Puhl RM et al., 2008). The findings of this study support there being no gender difference in weight-based stigma. However, we observed a significant difference in stress, with women experiencing more stress compared to men (Table 5).

**Table 5:** Two-sample *t*-test of stigma and stress by gender.

	**Male (n = 522)**		**Female (n = 824)**			
**Variable**	**M**	**SD**	**M**	**SD**	***t***	***p***
**Stigma**	0.087	0.105	-0.055	0.07	1.10	0.26
**Stress**	24.86	8.32	28.71	0.288	−8.29	0.000

Because socioeconomic status (SES) is important indicators of individual health, we additionally performed one-way ANOVA of stress and stigma by income and education to examine if education and income levels affect perceived stress and stigma. For stigma, no significant mean effect of education was observed, indicating that people perceive the same level of stigma regardless of their education level (Table 6). However, a significant mean effect of income was observed, with participants earning less than 5000 SR and those earning more than 15,000 SR experiencing less stigma compared to other income groups. These results reveal no surprises as many studies in the literature suggest that stigma impacts people regardless of educational background. For example, one *meta*-analysis assessing multiple studies on weight bias from healthcare providers found that prejudice exists among all populations regardless of education level (Moore CH et al., 2022).

**Table 6:** One-way ANOVA of stigma and stress by income.

	**Less than 5000 SR**		**5000**–**10000**		**10000**–**15000**		**More than 15,000**		**Prefer not to say**			
**Variable**	**M**	**SD**	**M**	**SD**	**M**	**SD**	**M**	**SD**	**M**	**SD**	**f**	**P**
**Stigma**	0.33	2.49	2.49	2.17	0.14	2.50	-0.13	2.21	-0.26	2.18	2.46	0.04
**Stress**	28.77	8.00	26.93	8.28	26.93	8.56	26.33	8.65	29.02	8.60	6.33	0.000

**One-way ANOVA of stigma and stress by education**														
	**Elementary**		**Secondary**		**High School**		**Associate**		**Bachelor’s**		**Graduate**			
**Variable**	**M**	**SD**	**M**	**SD**	**M**	**SD**	**M**	**SD**	**M**	**SD**	**M**	**SD**	**f**	**P**
**Stigma**	1.07	2.36	0.310	2.43	0.001	2.31	-0.380	2.08	0.040	2.32	0.047	2.44	0.77	0.57
**Stress**	26	5.65	30.2	3.61	28.04	8.49	27.25	7.56	27.18	8.53	24.98	8.90	2.83	0.01

Meanwhile, we found there to be a significant mean effect of education level in stress, in which participants that had completed higher education experienced less stress compared to those with lower education levels. Similarly, a statistically significant difference was observed in stress among participants of different income levels, with those having lower incomes scoring higher on stress; moreover, as income increases, stress level decreases (Table 6). Many studies have suggested that low socioeconomic status may cause people to live in poorer, more stressful settings, or may perpetuate their living in such areas (Brunello and D’Hombres, 2007, García Villar and Quintana-Domeque, 2009, Lundborg et al., 2007). Moreover, low socioeconomic status imposes a profound negative impact on psychological well-being and hence predisposes to psychological distress and depression (Tunceli K et al., 2006)^.^

Finally, despite the well-documented detrimental consequences of weight stigmatization on emotional and physical health, weight discrimination may constitute a major social and economic hurdle, especially in workplace and employment settings (Lundborg et al., 2007, Tunceli et al., 2006). In the present study, 24% of the participants reported having been stigmatized in employment settings and experiencing discriminatory hiring practices because of their weight, shape, or size. Parallel with our findings, existing evidence on weight discrimination in workplace and employment settings explicitly supports that obese individuals are less likely to be recruited, receive lower wages compared to thinner co-workers, and, in some cases, are unfairly terminated from their careers due to their high BMI levels (Brunello G and D’Hombres B, 2007). Furthermore, recent European studies have reported that weight stigmatization perpetuates discriminatory hiring practices among individuals with obesity. Specifically, it was estimated that every 10% increment in the average BMI correlates with a 1.86% drop in hourly earnings for a male employee and a 3.27% drop for a female employee (Lundborg P et al., 2007).

In Saudi Arabia, some employers may require job applicants to meet a specific weight or BMI criterion. For example, Saudi Aramco, the Saudi Arabian public petroleum and natural gas company, has been known to set limits including on weight and BMI, and applicants have in several instances been rejected due to not having the so-called “ideal body weight” (Akhbarr 24, 2013; Alrabyia Net, 2008). However, to our knowledge, no studies have investigated weight discrimination that occurs due to job requirements.

Many developed countries have instated a set of penalties against discriminatory acts based on personal characteristics such as race, age, color, sex, and disabilities. To date, weight discrimination has not been considered as a class protected from discrimination, although such discrimination may violate human rights in numerous instances (Australian Government: Attorney-General’s Department, 2014). Thus, people with obesity encounter major social and work-related inequities without clear legal penalties or policies to protect them against that discrimination.

## 5 Conclusion

To our knowledge, this is the first study to investigate weight-based stigma in Saudi Arabia. Parallel with international studies, our findings indicate a high prevalence of weight stigma in Saudi Arabia, which can have negative psychological implications for obese people. Therefore, we call for policies that that aim to protect overweight and obese people from discriminatory acts in the workplace, at school, and in advertisement and educational materials that portray obese people in a very negative, stereotypic way. Also, we call for more research to further understand weight stigma and its consequences on obese people in Saudi Arabia. We recommend focusing on the perceptions of healthcare providers towards obese people as doing so will provide deeper understanding of weight-based stigma in healthcare settings. In addition, different research designs such as qualitative research might be beneficial given the nature of the Saudi society. Finally, we suggest public health agencies, professionals, and advocates to develop and implement a public health intervention that focuses on improving the mental health, stress coping skills, and self-satisfaction of obese people.

Limitations.

This study has some potential limitations. One is that the cross-sectional design of the study may only demonstrate a temporal link and not a cause-effect relationship. The true link between outcome and exposure cannot be determined because both are examined at the same time. In particular, stress and stigma were examined once and although this examination implied a useful result, we believe a longitudinal perspective may reveal stronger and more comprehensive outcomes.

## Funding

This research did not receive any specific grant from funding agencies in the public, commercial, or not-for-profit sectors.

## CRediT authorship contribution statement

**Wafi Albalawi:** Conceptualization, Investigation, Project administration, Writing – original draft, Writing – review & editing. **Joud Albaraki:** Conceptualization, Investigation, Writing – original draft, Writing – review & editing. **Sereen Alharbi:** Conceptualization, Investigation, Writing – original draft, Writing – review & editing. **Nouf Ababtain:** Conceptualization, Investigation, Writing – original draft, Writing – review & editing. **Reema Alotaibi:** Conceptualization, Investigation, Writing – original draft, Writing – review & editing. **Ali Alsudais:** Conceptualization, Investigation, Writing – original draft, Writing – review & editing. **Jafar Jamjoom:** Conceptualization, Investigation, Writing – original draft, Writing – review & editing. **Meshal Alaqeel:** Conceptualization, Investigation, Project administration, Writing – original draft, Writing – review & editing.

## Declaration of Competing Interest

The authors declare that they have no known competing financial interests or personal relationships that could have appeared to influence the work reported in this paper.
